# Analysis of the Composition of Bromide Anion Oxidation Products in Aqueous Solutions with Different pH via Rotating Ring-Disk Electrode Method

**DOI:** 10.3390/membranes12090820

**Published:** 2022-08-23

**Authors:** Roman Pichugov, Dmitry Konev, Ivan Speshilov, Lilia Abunaeva, Mikhail Petrov, Mikhail Alexeevich Vorotyntsev

**Affiliations:** 1 EMCPS Department, Mendeleev University of Chemical Technology of Russia, 125047 Moscow, Russia; 2 Institute of Problems of Chemical Physics, Russian Academy of Sciences, 142432 Chernogolovka, Russia; 3 Frumkin Institute of Physical Chemistry and Electrochemistry, Russian Academy of Sciences, 119071 Moscow, Russia

**Keywords:** ring rotating disk electrode (RRDE), voltametric analysis, flow battery, bromide oxidation

## Abstract

We measured the ring collection coefficient of bromide anion oxidation products in a neutral and slightly alkaline medium on a rotating ring-disk electrode (glassy carbon disk, platinum ring) varying the following parameters: disk electrode rotation velocity, sodium bromide concentration, pH of the medium (in the range of 6–12), anode current on the disk, and the electroreduction potential of the bromide anion oxidation products on the ring. The data obtained are presented via dependences of the cathode ring current on the disk current ratio vs. the ring electrode potential. The analysis of the results was carried out by comparing the experimental polarization curves of the ring electrode with the data of cyclic voltammetry in model solutions to determine the electrical activities of various bromine compounds in positive oxidation states. We claim that the RRDE method could be used to obtain quantitative and qualitative data on the electrooxidation of bromide ions in neutral and alkaline solutions. For the most effective regeneration of the spent oxidizer, the values of pH > 10 and moderate concentrations of NaBr should be used.

## 1. Introduction

The global demand for high-energy power sources is growing annually due to the development of stationary and mobile electrical devices, the production volume of which is increasing rapidly. To meet the growing demand for energy sources for a wide range of applications, various energy storage devices have been developed to compete with fossil fuels. Among them, redox flow batteries (RFB) are one of the most promising candidates for stationary energy storage purposes, where energy is stored in the reduced or oxidized form of liquid chemical substances in the flowing media. The principal profit of the RFB operation, in contradistinction with solid state systems (e.g., lithium-ion batteries), is that it allows one to vary the energy capacity and the power density of the energy system in an independent way. This is achieved by varying the volume of posolyte (positive electrolyte) and negolyte (negative electrolyte) tanks and the electrode area inside the membrane electrode assembly (MEA), respectively. Moreover, the low cost of RFB devices, their prominent stability upon storage, their fire and explosion safety, the absence of toxic electrolytes or hazardous materials, and the absence of precious metal catalysts at the electrodes makes these systems perfect for outperforming competitors as energy storage devices for stationary applications.

Until recently, a fundamental drawback of most proposed flow batteries was their low power density (~0.2–0.4 W/cm^2^, for all-vanadium and iron-chromium redox flow batteries), which leads to high power costs and an increasing demand to find new high energy density electrolytes. With the discovery of hydrogen–bromine (H_2_-Br_2_) flow batteries, which have a power density of 1–1.5 W/cm^2^ and a round-trip efficiency of 91% at 1.6 A/cm^2^, these obstacles were overcome, and commercialization attempts in the USA and Israel resulted in grid-scale energy storage solutions [1,2,3,4,5,6]. Nevertheless, several fundamental problems remain unsolved, namely the moderate energy density and the toxicity and corrosive activity of bromine. In order to increase the energy density of bromine, substantial progress has been made exploring new multi-electron oxidizers. Recently, bromate oxidizer has attracted interest as a replacement for bromine, due to its extremely high charge density and energy densities. This is due to the very high solubility of bromate lithium salts and its multi-electron reduction process, which uses six electrons for its rapid transition to bromide in strong acidic conditions [7,8]. The reported bromate RFB reaches a specific power of 1 W/cm^2^ for 1 M H_2_SO_4_ [9], yet there is an evident lack of research on the regeneration process of the utilized oxidizer in the form of acidic bromide solution compared to the initial bromate solution. Therefore, this research aims to produce a complete energy cycle for a system of this nature. The general reaction of the bromate formation can be written as [10]:

Br^−^ + 3H_2_O = BrO_3_^−^ + 6H^+^ + 6e^−^ (1)

There are two known ways to perform this conversion:

- Heterogeneous oxidation of bromide (during the stage of formation of bromine) to bromate, which can be realized in an acid medium.
- Homogeneous reaction of disproportionation of bromine in a slightly alkaline medium.

The formation of bromate in a neutral or slightly alkaline medium was experimentally demonstrated. Pavlovic et al. states that during the electrochemical formation of bromate, the current efficiency cannot be above 67%, as one third of the current is used for oxygen evolution [11]. The systematic study of bromide oxidation with bromate formation on boron doped diamond electrodes (BDD) was performed by Bergman [12]. Vacca et al. indicated that the main mechanism of bromate formation is the oxidation mediated by hydroxyl radicals and oxygen radicals [13]. Cettou et al. obtained contradictory results regarding the current efficiency in bromate formation. They reported that 90% current efficiency was required for a primary reaction. However, from the thermodynamic point of view, favored reactions should proceed at a lower potential. According to Cettou [10], the standard potential for the anodic formation of bromate is approximately 1.45 V, whereas the standard potential for bromine discharge is only 1.1 V. In addition, in many studies on the electrolysis of bromide to bromate, low concentrations of bromide are used, and electrolysis is carried out at potentials beyond the electrochemical stability window of aqueous electrolytes [12].

Therefore, in slightly alkaline solutions, the oxidation of hydroxyl ions and the formation of bromate from hypobromite and bromide are thermodynamically preferred. When all bromate is formed by the chemical reaction, the resulting current efficiency can reach 100%. To favor this approach, neutral or alkaline solutions should be preferred when the primary method of bromate formation is the reaction of hypobromous acid disproportionation [14]. Several research groups demonstrate this; Cettou [10] used the Ti/RuO_2_ electrodes to show how bromate is formed upon the oxidation of hypobromite by hypobromous acid. Pavlovic et al. stated that the current efficiency of bromate formation reaches 98–99% in a pH range between 8.5 and 9.5 on DSA electrodes [11]. Nevertheless, the overall reaction is of the third order, and it is 10 times faster than the chlorate formation at the same temperatures [10].

To form a closed energy cycle for the bromate RFB, it is necessary to perform regeneration of the spent oxidizer and to consider a further optimization parameter, round-trip energy efficiency. To obtain sufficient values for cycle efficiency, the optimal method of regeneration (electrooxidation) should be determined. The key issues in bromide oxidation in neutral and alkaline media are the current yield of the desired product and the composition of the near-electrode layer. A traditional electrochemical method for solving this issue is by using a rotating ring-disk electrode (RRDE). For example, in the work by Johnson, an RRDE was used to study the oxidation of bromide on a platinum electrode in 1 M sulfuric acid [15]. On one of the electrodes (on the Pt disk), bromine was generated after the application of anode current. On the second electrode (Pt ring), products of bromide oxidation were registered in the cathode part of this process. It was shown that the oxidation of bromide in these conditions results in the formation of not only bromine, but also hypobromous acid. However, no references exist regarding the application of the RRDE method to neutral and alkaline media with high bromide concentrations.

Therefore, the existing experimental data is not enough to analyze the combination of electrochemical and chemical stages of bromate regeneration. As a result, it is necessary to determine whether it is possible to obtain the data for a combination of stages using the rotating ring-disk electrode method. There is a lot of information on the oxidation of bromide to bromate, although rigorous analysis of the data has not been carried out. The voltammetric analysis of the ring facilitates the study of the rate of disproportionation of bromine and its various products. To achieve this goal, we performed a thorough analysis of bromide oxidation in buffer solutions at different pH levels using a rotating ring-disk electrode.

## 2. Experimental

### 2.1. Chemicals

Sodium bromide (99%, Laverna, Moscow, Russia) and triple-distilled water were used for electrolyte preparation. The required amount of sodium bromide powder was added to the prepared buffer solution at a predetermined pH. All experiments were performed at 2 concentrations of bromide: 0.5 M and 1 M. High concentrations of bromide ion are studied due to their potential use in RFB.

The sodium bromate (99%, Labteh, Moscow, Russia) was used as received. Solutions of HOBr were prepared through the reactions of bromine disproportionation in alkaline medium, i.e., acidic bromine solution was neutralized by 2.5 M NaOH (99%, Laverna, Russia).

Buffer solutions were prepared according to the procedure described in the literature [16]. Buffer solutions with pH 10.5, 10, and 9.5 were prepared by mixing different amounts of 0.1 M aqueous sodium hydroxide solution (NaOH) (Labteh, Moscow, Russia) and 0.05 M aqueous sodium tetraborate solution (Na_2_B_4_O_7_·10H_2_O) (Labteh, Moscow, Russia) to give the required pH. Buffer solutions with pH 7.5, 7.02, 6.5, and 6 were prepared by mixing different amounts of 1/15 M aqueous solution of KH_2_PO_4_ (Labteh, Moscow, Russia) and 1/15 M aqueous solution of Na_2_HPO_4_ (Labteh, Moscow, Russia) to give the required pH. A buffer solution of 0.1 M NaOH was used with a pH of 12.5.

When the buffer solution was prepared, a pH meter ANION 4100 (NDT-Group, Novosibirsk, Russia) was used to determine the pH.

### 2.2. Procedures

Electrochemical measurements were performed at room temperature using an Autolab PGSTAT302N potentiostat/galvanostat. A rotating ring-disk electrode with a rotation speed control Pine MSRX (Pine Research Instrumentation, Durham, NC, USA) in a double-walled three-electrode cell (volume 200 mL) was used for all electrochemical measurements. A working electrode RRDE consisted of a glassy carbon (GC) disk (diameter 5 mm) and Pt ring (outer diameter 7.5 mm and inner diameter 6.5 mm). Pt wire was used as a counter electrode and Ag/AgCl was used as a reference electrode. It was filled with saturated KCl solution and was in electrical contact with the solution through a salt bridge filled with 1 M H_2_SO_4_.

To prepare for each measurement, the working electrode was gradually polished with SiC paper of decreasing abrasive grain size and a diamond suspension of 1/4 mm (Escil, Chassieu, France).

### 2.3. Ring Collection Coefficient Measurements

For systematic study of the bromide anion oxidation process, and in order to understand its kinetics, products, and composition in a qualitative and quantitative manner, the following experimental procedure was employed.

Prior to the measurement of the ring collection coefficient, cyclic voltammograms were recorded for each composition to determine the range of potentials and currents when the primary reaction of bromide oxidation occurs without simultaneous intensive oxygen evolution. Among the studied solutions, this value was defined as the most alkaline solution. The maximum current was determined to be 10 mA in 0.1 M NaOH and was used for all buffer solutions.

The following procedure was used for all NaBr solutions studied with different pH levels, from 6 to 13, and a constant current of 10 mA was applied to the GC disk. The GC electrode was selected due to its 10 times lower current densities compared to the Pt ring, recorded by scanning CVs of buffer solutions in the case of neutral and alkaline pH.

Under the application of current, the electrochemical oxidation of bromide to bromine on the surface of the GC disk occurred. The potential of the Pt ring took specific values. In this case, the curve of the current decay was registered on the ring. Each value of ring potential remained unchanged for 20 s to obtain a steady-state value of current. The integration value for the final 2 seconds was recorded as a current value on the Pt ring, corresponding to this potential. The ring potential values varied from +0.65 V to −0.9 V. The potential range of the ring was chosen to be far from the equilibrium potential of the bromide–bromine redox couple (i.e., bromine generated on the disk would be reduced on the ring close enough to the diffusion limited regime). Such a wide range of potentials is studied due to the possibility of the reduction of other bromine containing products, for example HOBr (or BrO^−^), as described in several articles [15].

Similar dependencies were measured for other bromine containing species to determine the applicability of the proposed experimental setup.

The ratio of the value of the measured current on the Pt ring to the applied current on the disk is called the “ring collection coefficient”. This value was measured for 2 different rotation speeds–200 rpm and 5000 rpm–modeling extremely low and high rates of solution supply to the electrochemical reactor and, consequently, the transfer rates of reaction products from the disk to the ring.

To determine the maximum value of the ring collection coefficient and to control the conditions of its constancy, an experiment of electrochemical bromide oxidation was performed in 1 M H_2_SO_4_ solution, where the oxidation product of bromide (bromine) is thermodynamically stable according to the Pourbaix diagram [17,18]. The obtained value of the ring collection coefficient in this case was 0.25 ± 0.1. The theoretical formula [19] for the geometry of RRDE estimates 0.26, indicating an agreement with the experimental data.

Moreover, to eliminate the influence of a side reaction on the ring electrode through the electroreduction of dissolved oxygen, the experimental algorithm was also supplied with the measurements and substractions of the background currents on the Pt ring, obtained under the application of zero current to the GC disk.

## 3. Results and Discussion

### 3.1. Electrochemical Activity of Various Bromine-Containing Species

To study the oxidation of the bromide process in neutral and alkaline solutions, it’s current efficiency, and the composition of the near-electrode layer via the rotating disk electrode method, the electrochemical properties of bromine-containing compounds were studied.

#### 3.1.1. Electrochemical Oxidation of Bromide Anion

**Figure 1:**
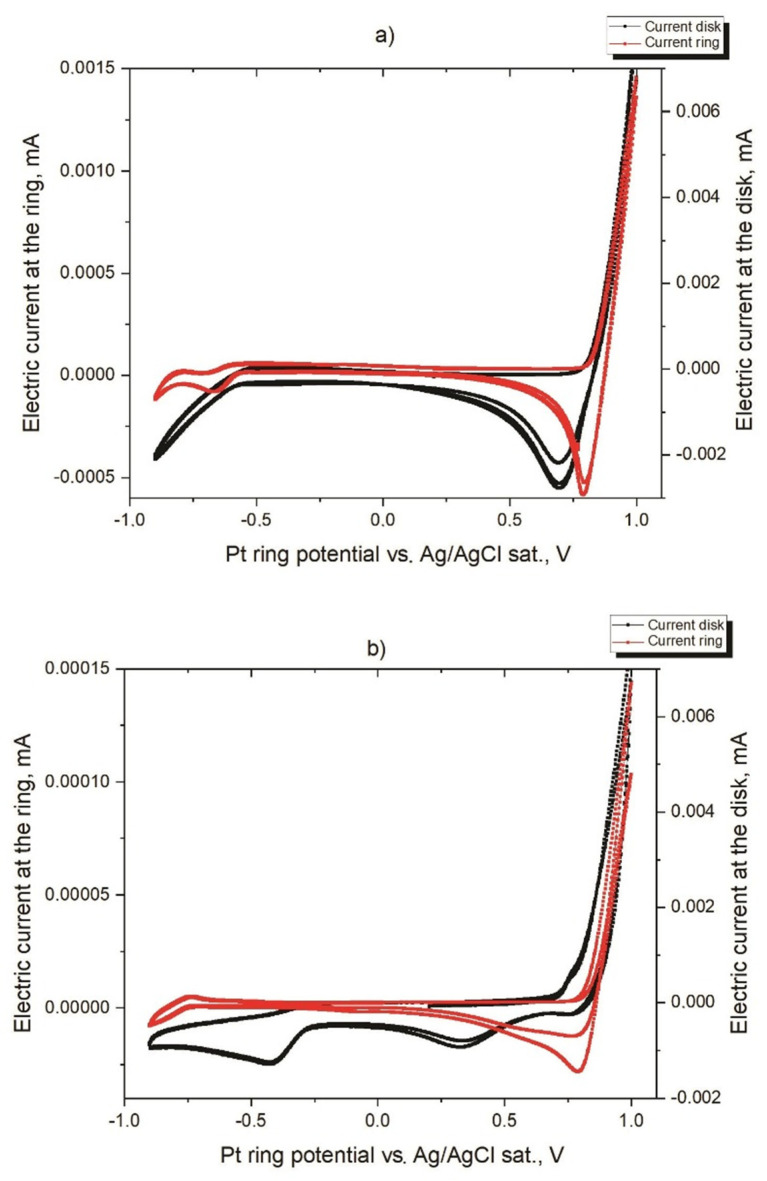
Cyclic voltammograms of 0.5 M NaBr solutions measured at scan rate 50 mV/s at different pH values of buffer solutions at rotating disc and ring: (**a**) pH = 7.02 (**b**) pH = 9.93. Simultaneously, data for these solutions in the form of the ring rotating disc which collected current dependencies are presented in Figure 2.

To determine the range of potentials and currents when the primary reaction of bromide oxidation occurs without simultaneous intensive hydrogen or oxygen evolution, cyclic voltammograms of NaBr solutions were recorded using electrolytes with different pH values (see Figure 1).

As the bromide electrooxidation process does not involve protons, the redox peak does not depend on the pH value and is at the cathodic area near +0.8 V. As there is no hydrogen evolution on the left side of Figure 1 until at least 0.8 V, the available potential window for the experimental setup is approximately between −0.8 V and 0.8 V.

Br_2_ + 2e^−^ = 2Br^−^ (2)

Additionally, by imposing a constant 10 mA disk current value, we produced a chemically equivalent amount of bromine to collect at the Pt ring, registering dependencies of the current values at the ring vs. its potential for alkaline (pH = 9.93) and neutral (pH = 7.02) conditions. Such a setup allowed us to examine the possible occurrence of a homogeneous chemical step, e.g., bromine disproportionation in alkaline medium:

Br_2_ + 2OH^−^ = BrO^−^ + Br^−^ + H_2_O (3)

**Figure 2:**
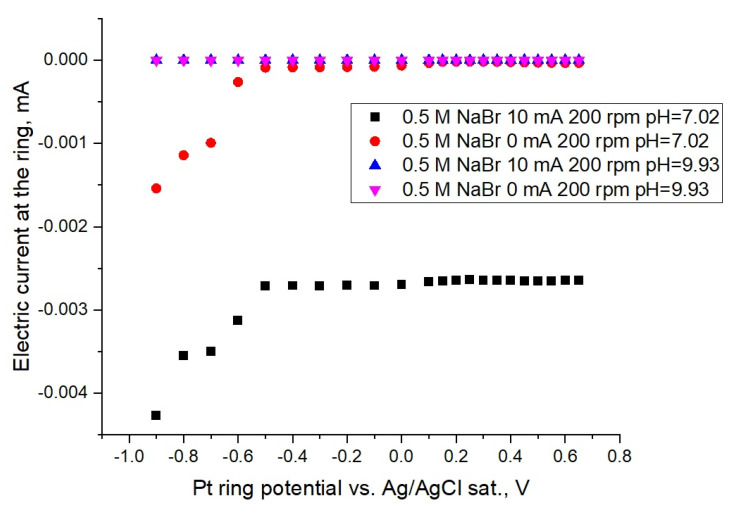
The Pt ring collected current dependencies on the potential for 0.5 M NaBr, at different pH of solutions (black and red points correspond to pH = 7.02, violet and blue points correspond to pH = 9.93) and under stationary conditions while rpm = 200. Simultaneously, data for these solutions in the form of cyclic voltammograms are presented in Figure 1.

Simultaneously, we used a cyclic voltammograms analysis (see Figure 1) to perform the ring rotating disc electrode experiments (see Figure 2). According to Figure 2, the constancy of the collected current in the whole interval of potentials witnesses the absence of chemical transformation in the neutral solution, while bromine species move from the disk to the ring surface and the diffusion limited regime takes place.

In the alkaline solution, no current is observed, as the bromine disproportionation occurs via a homogeneous reaction. To verify the experimental setup for both cases, control experiments were performed, with no current imposed on the disk and the absence of current at the ring.

#### 3.1.2. Electrochemical Activity of NaBrO_3_ on Pt Ring in Solutions with Various pH Values

**Figure 3:**
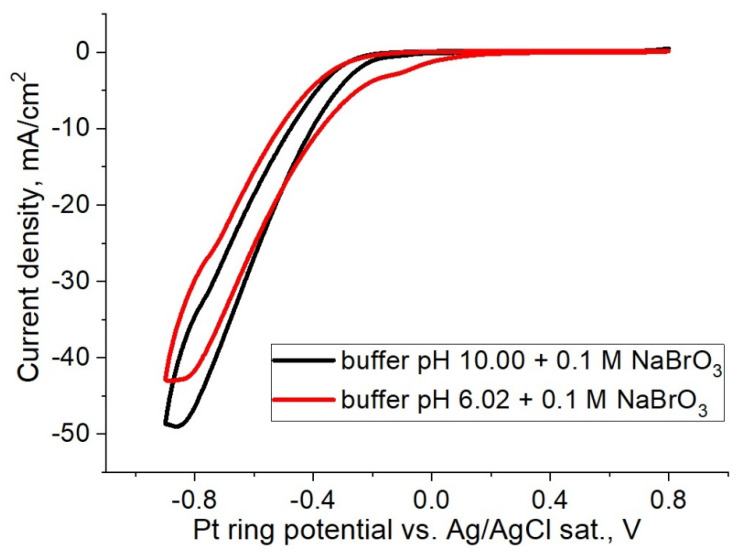
Cyclic voltammograms of NaBrO_3_ in buffer solutions with pH = 6.02 and pH = 10, recorded on a stationary platinum electrode at a scan rate of 50 mV/s.

To determine the electrochemical activity of bromate anions, the potential range was limited in the anodic region by 0.8 V to prevent the formation of a bromide/bromine redox couple. Figure 3 demonstrates CVs of NaBrO_3_ in the solutions with different pH levels, indicating that bromate anions exhibit electrochemical activity at much more negative potentials than the bromide/bromine redox couple and that the process is independent of the pH of the electrolyte.

**Figure 4:**
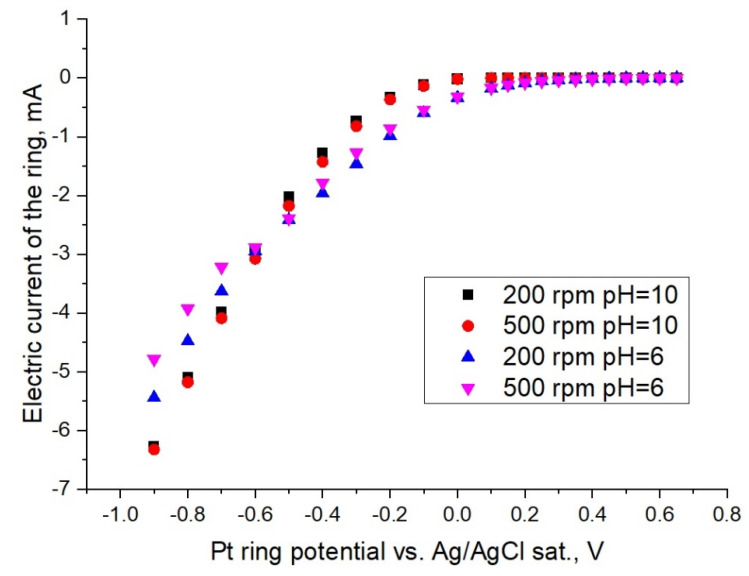
The Pt ring collected current vs. the potential dependence under stationary conditions for 0.1 M NaBrO_3_ in various buffer solutions at RRDE different rotation velocities.

The ring current vs. the potential dependence of NaBrO_3_ is shown in Figure 4 for different pH and rotation rates of the RRDE (while no current is imposed at the disc). In the potential range of 0.6 V to 0 V, no current on the ring was registered. Furthermore, the cathode current on the ring increases proportionally as the potential shifts to more negative values.

The polarization curves showing clear similarities in Figure 3 and Figure 4 are noteworthy. In the negative potential region under 0 V, the observed cathodic waves are attributed to the discharge of bromate anions onto the platinum electrode. These observed currents are apparently associated with the direct bromate anion reduction. The possibility of a redox-mediated bromate electroreduction should be excluded (EC” mechanism) due to the absence of the strongly acidic medium, which is essential for such an autocatalytic process [7,20], so that the observed current can be associated with the discharge of bromate anions.

#### 3.1.3. Electrochemical Activity of HOBr (OBr^−^) on the Pt Ring

The electroreduction of bromine (+1) compounds on platinum has been widely studied [15]. The equilibrium potential of this process in solutions of 1 M H_2_SO_4_ (pH = 0) was found to be 1.34 V compared to the standard calomel electrode. As our experimental conditions are different, we performed our control estimation based on this value.

**Figure 5:**
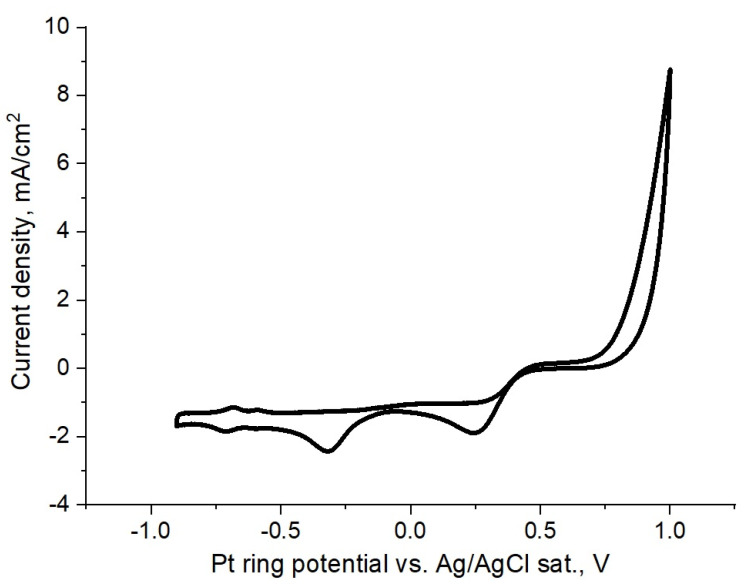
Cyclic voltammograms of 0.1 M OBr^−^ recorded at 50 mV/s on the stationary platinum electrode. pH = 11.66.

Figure 5 shows cyclic voltammograms of HOBr in solutions with an alkaline pH. Different pH values were obtained depending on the amount of alkali added to neutralize the initial acidic bromine solution. When comparing the previously obtained curves for bromates, a peak in the +0.3 V region is presumably associated with the electroreduction of HOBr to Br^−^.

**Figure 6:**
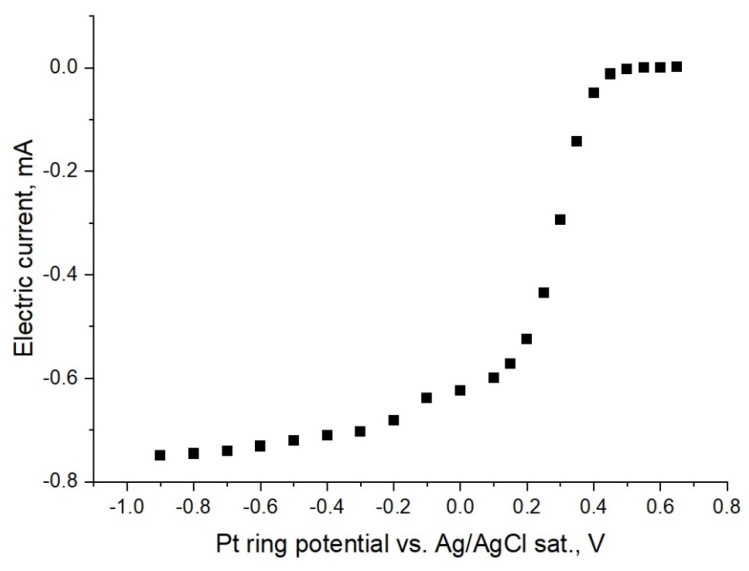
The Pt ring collected current vs. the potential dependence under stationary conditions for 0.1 M OBr^−^. pH = 11.66, rpm = 200.

Figure 6 shows the dependence of the collected current on the potential of the platinum ring for BrO^−^. This dependence shows that at a potential value below 0.4 V there is a noticeable increase in the current on the ring, indicating that the electroreduction of the hypobromite anion occurs at this potential. The current increase continues, reaching a certain limiting value at −0.1 V. It then increases slightly due to the superposition of two processes occurring simultaneously in accordance with the peak points in Figure 5—one starting at 0.1 V, another at −0.3 V.

The presented experimental data suggests the possibility of using the RRDE method in the electrooxidation reaction of bromide ions to obtain quantitative information on the kinetics of this complex process. In particular, the conversion rate of the primary product of bromide oxidation (molecular bromine) into an oxygen-containing bromine compound (hypobromite ion, or its undissociated form HOBr) and bromate ion BrO_3_^−^ can be estimated based on the experimental dependencies of current collection coefficients from the potential of the Pt ring electrode. This possibility is based on the difference in potentials of electroreduction waves in these components:

- Br_2_ reacts in the whole potential range, including the region above +0.5 V in the diffusion-limited regime.
- BrO^−^ ion is reduced in the potential range near +0.5 V, reaching the diffusion limit near +0.1 V.
- Finally, BrO_3_^−^ begins to react near +0.1 V, with a gradual increase in the reaction at more negative potentials.

### 3.2. Ring Collection Coefficients at Different pH Values

As shown in the previous sections, the obtained experimental data on collected current values on the Pt ring are an integral characteristic, as both molecular bromine and products of bromine disproportionation with a higher oxidation state can be reduced on the ring. In addition, the charge balance may be maintained by the constancy of the ring current (and, consequently, collected current values) regardless of the occurrence of the disproportionation reaction and its rate in the case of identical electrochemical activity of all bromine forms (0, +1, +5) on the ring electrode. However, the obtained experimental data, and several experimental works in the literature [21], indicate the significant overvoltage for the electroreduction of bromine compounds with oxidation states +1 and +5, which should lead to the separation of corresponding product reduction waves. This makes it possible to monitor the near-electrode concentration of molecular bromine in the certain range of potentials. To verify this, the following studies were performed.

In this section we compare the dependencies of current collection efficiencies on the ring potential at different parameters of the studied system. This is undertaken in order to evaluate their effect on the composition of the oxidation products of the bromide ion considering the subsequent chemical stages. The following dependences are present for the specific NaBr concentration, the current on the GC disk, and the rotation speed of RRDE. The data allows for analysis of the effects of the pH solution, the concentration of bromide passed through the GC disk, and the rotation speed of the RRDE.

The ring collection efficiency was determined as follows:

(4) $$N=\frac{I_{\left(\mathrm{ring}\right)}-I_{0\left(\mathrm{ring}\right)}}{I_{\left(\mathrm{disc}\right)}}$$

where *I*_(ring)_ is the current registered on the Pt ring when the current is applied to the GC disk, *I*_0(ring)_ is the current registered on the Pt ring when zero current is applied to the GC disk, and *I*_(disc)_ is the value of the current applied to the GC disk.

**Figure 7:**
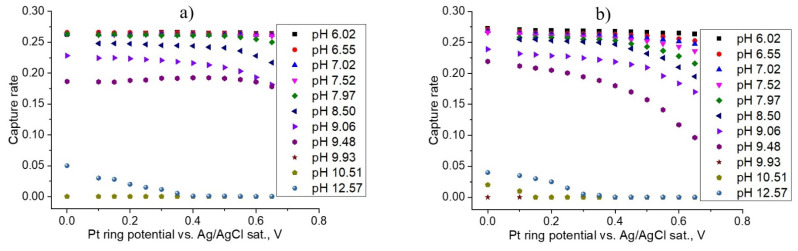
Ring collection efficiencies on the Pt ring under the applied 10 mA on the GC disk for 0.5 M NaBr solutions at different pH levels. The graphs are presented for two rotation rates of the RRDE: (**a**) 200 rpm and (**b**) 5000 rpm.

**Figure 8:**
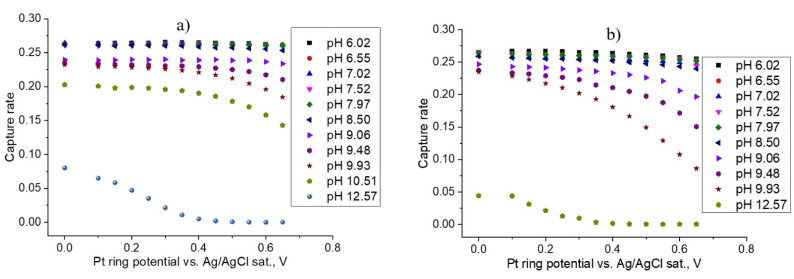
Ring collection efficiencies on the Pt ring under the applied 10 mA on the GC disk for 1 M NaBr solutions at different pH levels. The graphs are presented for two rotation rates of the RRDE: (**a**) 200 rpm and (**b**) 5000 rpm.

Figure 7 and Figure 8 demonstrate the dependencies of the ring collection efficiencies for 0.5 M NaBr and 1 M NaBr, respectively.

The presented data shows that the RRDE method allows quantitative information on the kinetics of this complex process to be obtained, which includes the electrochemical oxidation of bromide to bromine and subsequent chemical stages, involving bromine disproportionation in a neutral and alkaline medium. As it was demonstrated in previous sections, the bromine is reduced in the whole range on the Pt ring potential in Figure 7 and Figure 8. Moreover, the reduction of BrO^−^ (or the undissociated form HOBr) occurs in the potential region from +0.5 V, reaching the diffusion limit at +0.1 V. From this point, the current collection efficiency in the potential region from 0 V to 0.7 V should be equal to its maximum value if other bromine-containing compounds are not formed.

In some cases, the current collection efficiency does not reach its maximum value of 0.26, indicating that the bromate formation occurred in an alkaline media (pH > 9). Based on this assumption, we demonstrated that at specific pH levels only bromate is formed in the system, revealing striking disagreement with the reported experimental data by [10,15].

At high concentrations of NaBr, the disproportionation reaction occurs at pH > 9. When the NaBr concentration increases, the rate (degree) of bromine disproportionation decreases at the same pH value due to the possible formation of Br3^−^. By comparing two different rotation rates for the RRDE, we can state that bromate is not formed at a high rotation rate (5000 rpm) of RRDE.

As a result, the obtained experimental data indicate that the chemical reaction of bromine disproportionation in an alkaline medium is rather fast. Therefore, the disproportionation reaction can be used to regenerate the spent oxidizer.

## 4. Conclusions

The RRDE method could be used to obtain quantitative and qualitative data on the electrooxidation of bromide ions in neutral and alkaline solutions. We showed experimentally that the chemical reaction of bromine disproportionation in an alkaline medium leads to bromate formation and that the reaction proceeds quickly, which does not correspond to the known literature. Thus, the disproportionation reaction can be used to regenerate the spent oxidizer in a hydrogen–bromate redox flow battery. For the most effective regeneration of the spent oxidizer, values of pH > 10 and moderate concentrations of NaBr should be used.

## Data Availability

Not applicable.
